# Wedge Resection and Nail Groove Reconstruction With Hanging Thread Knot in the Treatment of Onychocryptosis

**DOI:** 10.1111/jocd.16585

**Published:** 2024-09-30

**Authors:** Yonghong Hao, Xiaofang Zou, Zigang Zhao, Liyuan Xing, Chengxin Li

**Affiliations:** ^1^ Department of Burns and Plastic Surgery People's Liberation Army Air Force General Hospital Beijing China; ^2^ Department of Dermatology The PLA General Hospital Beijing China; ^3^ Department of Dermatology First Affiliated Hospital of Chinese PLA General Hospital Beijing China

**Keywords:** anti‐infective, classification, onychocryptosis, surgery

## Abstract

**Background:**

Onychocryptosis is characterized by the nail plate penetrating the lateral nail fold, resulting in varying degrees of infection and deformity. Standardized treatment protocols for onychocryptosis, particularly in Stages IIb, III, and IV, have not been universally established, highlighting the urgent need for the development of effective interventions.

**Objective:**

To evaluate the effectiveness and safety of wedge resection and nail groove reconstruction using the hanging thread knot for the treatment of onychocryptosis.

**Methods:**

At our hospital, a total of 155 patients with onychocryptosis in Stages IIb, III, and IV underwent treatment. Wedge resection and nail groove reconstruction with the hanging thread knot were applied based on the severity of deformity and infection for treating onychocryptosis. All patients received perioperative systematic and topical anti‐infective treatments. Follow‐ups conducted over a period of 2–6 months assessed postoperative rehabilitation and complications.

**Results:**

The cure rate reached 95%, with a low recurrence rate of 5%. Recurrence, observed in eight patients, was attributed to various causes: three due to improper trimming, three related to trauma, one associated with obesity, and one due to incomplete matrix resection. All eight patients achieved complete recovery through health guidance and secondary surgery. Satisfaction results were reported during the 2–6 months follow‐up period. Although 10 patients experienced secondary local infections, all achieved complete recovery following active treatment.

**Conclusion:**

Wedge resection and nail groove reconstruction with the hanging thread knot prove to be an effective and safe method for treating onychocryptosis.

## 1 Introduction

Onychocryptosis is a pathological nail condition characterized by pain, erythema, swelling, discharge, and granulation tissue formation around the nail fold, resulting in significant impairment of daily activities [1, 2, 3]. Its etiology encompasses anatomical and physiological mechanisms as well as comorbidities and external factors such as trauma, inappropriate trimming, poor hygiene, and ill‐fitting footwear [1, 4, 5]. This condition is categorized into Stages I through IV based on severity [6], with non‐surgical approaches recommended for Stages I and IIa while surgical interventions are indicated for Stages IIb through IV. The selection of surgical techniques may yield varying outcomes and impacts on patients. Following an extensive review of literature and clinical cases, wedge resection along with nail groove reconstruction using the hanging thread knot has demonstrated efficacy in managing Stage IIb through IV onychocryptosis.

## 2 Materials and Methods

### 2.1 Patients

A retrospective analysis was conducted on 155 patients (190 ft) who underwent wedge resection and nail groove reconstruction with the hanging thread knot at our hospital's dermatology department from January 2016 to March 2022. The cohort consisted of 110 males (135 sides) and 45 females (55 sides), aged between 7 and 65 years, with an average age of 25 years. The duration of the disease ranged from 4 months to 5 years. Among them, 109 patients (142 sides) were in Stages IIb and III, while 46 patients (48 sides) were in Stage IV. Exclusion criteria included congenital phalangeal deformity, severe circulatory disorders, diabetes, pregnancy, impaired wound healing, and known coagulopathy or uncooperativeness. Informed consent was obtained from all patients, and the study received approval from our hospital's Ethics Committee.

### 2.2 Surgical Technique

**FIGURE 1:**
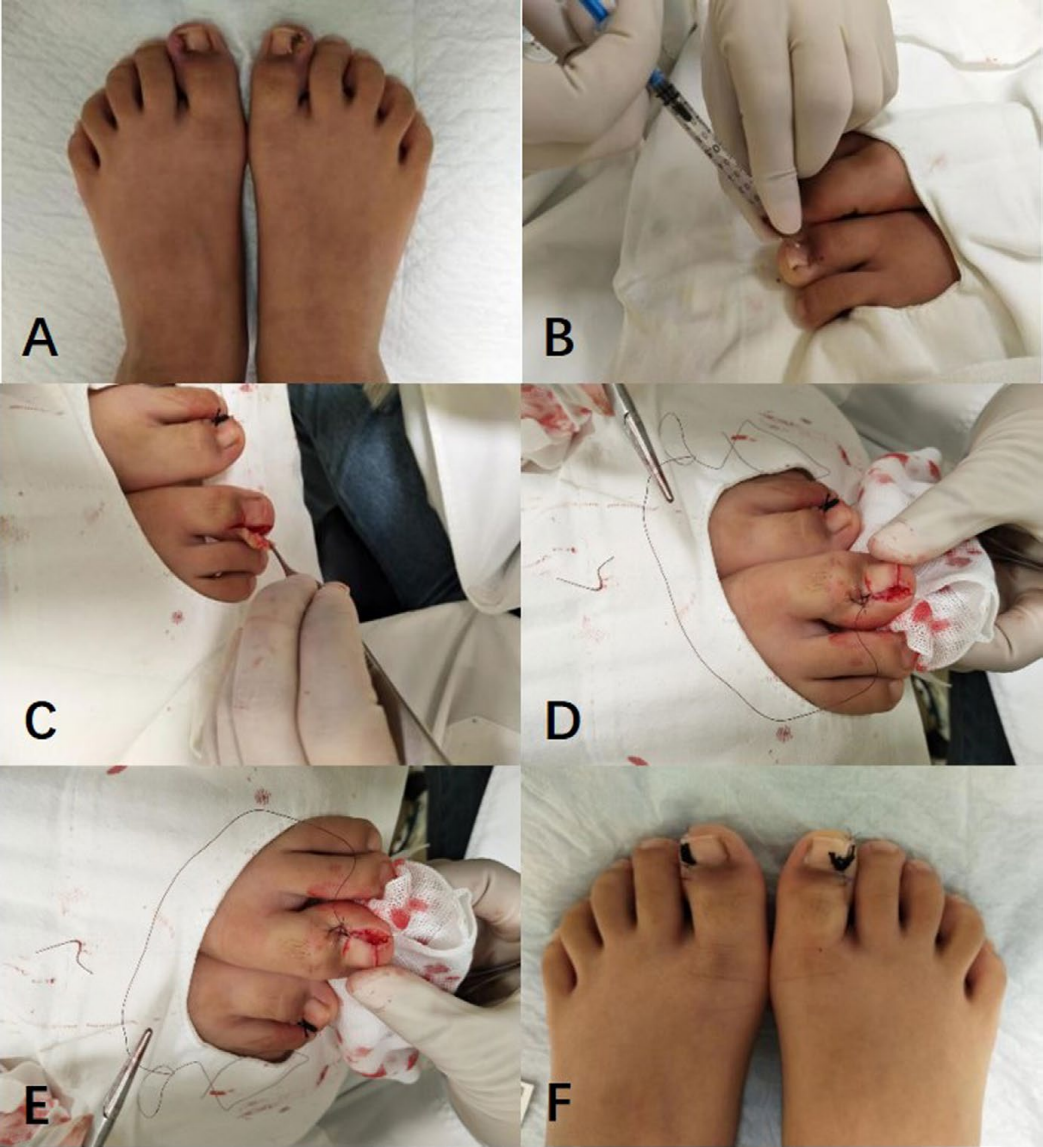
Surgical technique: (A) Management of Stage IIb and III onychocryptosis. (B) Administration of local anesthesia. (C) Excision of granulation tissue in a wedge‐shaped manner. (D) Fixation of the nail fold with periosteal tissue. (E) Reconstruction of the nail sulcus. (F) Immobilization of the suture knot at the lateral edge of the toenail root.

Following local anesthesia (Figure 1), the embedded toenails were initially excised 1–3 mm from the lateral edge, concomitantly removing hyperplastic granulation tissue and the pathological nail plate in a wedge‐shaped manner. Subsequently, the fresh nail fold was secured and sutured to the periosteum of the lateral edge of the toenail, achieving reconstruction of the nail groove. Finally, a 2‐0 nylon thread knotted suture was anchored beneath the lateral deck of the root of the affected side toenail to rectify its growth orientation.

### 2.3 Perioperative Management

Prior to surgery, each patient provided informed consent, and received oral antibiotics and analgesics during the perioperative period. Surgeons provided instructions for timely dressing changes and conducted evaluations of wound healing parameters including pain, edema, and signs of infection.

## 3 Result

Satisfactory outcomes were observed during the 2‐ to 6‐month follow‐up. Among the 10 patients who experienced wound infection, 6 cases were attributed to incorrect dressing change procedures, 1 case was linked to nylon line rejection, and 3 cases resulted from trauma and strenuous activity. Professional dressing changes effectively facilitated healing in these instances. Onychocryptosis recurred in eight cases: two due to improper toenail trimming, three related to football‐related trauma, one associated with obesity, and two as a result of incomplete matrix resection. Notably, patients did not express dissatisfaction with the cosmetic results of hallux.

## 4 Discussion

**FIGURE 2:**
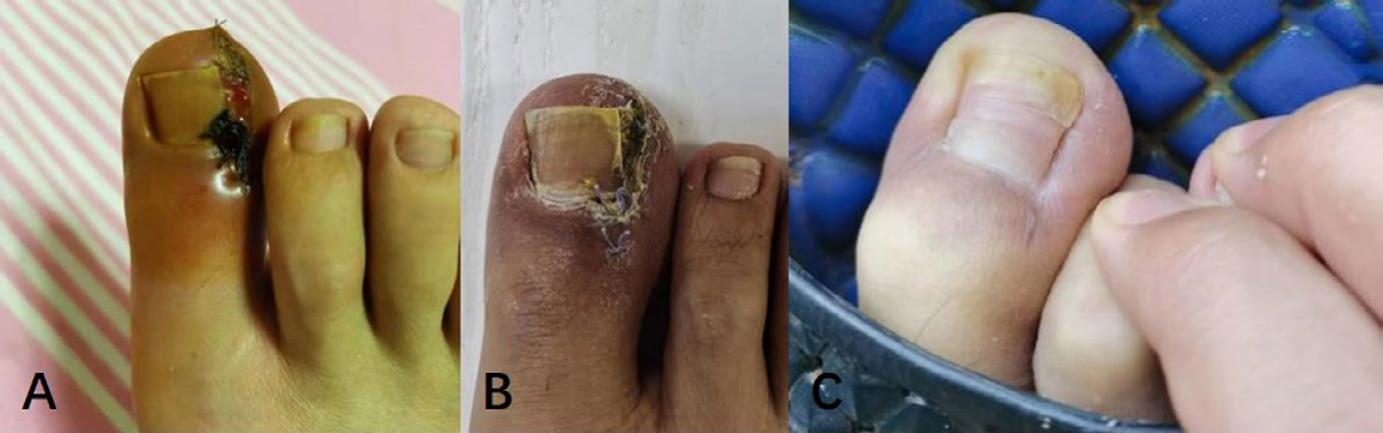
Surgical Technique: (A) Illustration depicting suture rejection reaction. (B) Removal of sutures and regular dressing changes. (C) Follow‐up photographs taken at 2 months post‐surgery.

**FIGURE 3:**
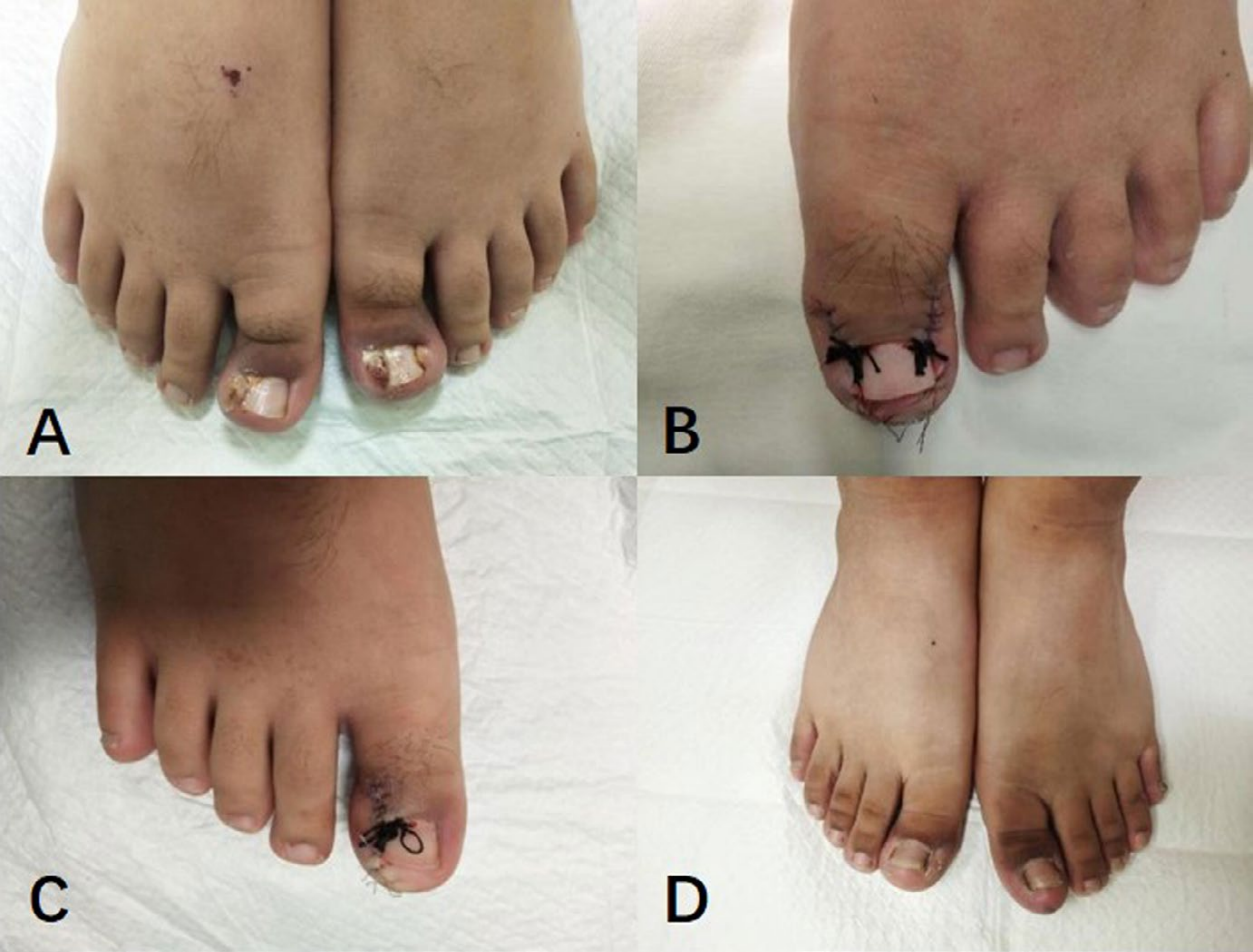
Suture rejection reaction: (A) Onychocryptosis in Stages IIb, III, and IV. (B) Postoperative assessment of the left foot. (C) Postoperative assessment of the right foot. (D) Follow‐up evaluation at 2 months post‐surgery.

Onychocryptosis, commonly known as an ingrown toenail, is a prevalent pathology that affects the general population [1]. The severity of symptoms varies, with a preference for the hallux and its peroneal edge in young males [7, 8]. Contributing factors to onychocryptosis include anatomic abnormalities, biomechanical alterations, improper nail trimming, inappropriate footwear, obesity, and secondary bacterial or fungal infections [9]. Repeated stimulation from various factors disrupts the migration of the nail bed to the nail wrinkle epithelium, interrupting the process. Proliferative granulation tissue covered by epithelium not only hinders abscess drainage but also locally forms scars that contribute to wrapping growth of the nail bed to the phalanges [10].Chronic inflammation and spicule irritation further exacerbate hyperplasia of the granulation tissue, thereby increasing the risk of osteomyelitis [11]. The selection of treatment depends on the etiology and severity of onychocryptosis. Non‐surgical options, such as nail fold taping, acrylic artificial nails, nail packing, dental floss, gutter technique/sleeve technique, nail tube splinting, nail brace technique, and phenol‐alcohol technique are effective for milder cases (Stages I and IIa) [12, 13, 14, 15, 16]. Surgical interventions, such as the Winograd technique, esthetic reconstruction, and phenol total matrixectomy, are recommended for moderate or severe cases (Stages IIb, III, and IV respectively) [17, 18]. Surgical approaches entail excision of the pathological nail plate and hypertrophic inflammatory granulation tissue [19]. The surgical principles involve rectifying structural deformities of the nail and restoring normal morphological and physiological characteristics of the nail folds (Figure 2). In our investigation, the use of a hanging thread knot was simultaneously employed to rectify the toenail's growth trajectory, ensuring comprehensive reconstruction of the nail groove and aligning the toenail's growth direction while preserving the integrity of the normal toenail. Despite thorough preoperative cleansing, contamination of fingernails by *Staphylococcus epidermidis*, which is the predominant microorganism residing on fingernails, persisted. Consequently, some researchers propose that administering oral and topical antibiotics around the perioperative period can mitigate the risk of infection [1, 9, 19]. Surgical interventions are not recommended in the presence of infection [20, 21]. In clinical practice, we offer patients guidance on antibiotic administration and wound management during the perioperative period. After cleansing the wound with hydrogen peroxide solution and povidone iodine, topical antibiotic ointment is applied to the wound twice daily. It is recommended to schedule surgery after effectively managing infectious and swelling symptoms. In cases of severe hallux swelling, clinical experience indicates that hypertonic saline wet compress is an effective intervention. Surgeons take into consideration not only the severity of onychocryptosis, infection, and granulation tissue but also assess the associated risks and benefits, explore alternative treatments, and consider patient preferences [22]. Various surgical interventions, such as radical excision of the nail folds, wedge resection, and nail avulsion with chemical matricectomy using trichloroacetic acid or phenol, have been documented in moderate and severe cases [3, 23]. Despite these interventions, the literature reports high recurrence rates [24]. Based on both the existing literature and our specific case characteristics, we have implemented wedge resection and thread‐ligating therapy for Stage IIb, III, and IV onychocryptosis (Figure 3). During the procedure, particularly in cases of bilateral onychocryptosis, special attention is devoted to safeguarding the neurovascular plexus in order to prevent necrosis of the distal phalanx.

Inadequate resection of the methyl on the lateral aspect of the toenail may result in ectopic nail formation [25]. If delayed wound healing is observed several weeks post‐treatment, considerations should encompass potential rejection of sutures (Figure 3), osteomyelitis, or local squamous carcinoma and other malignant neoplasms. Typically, the residual suture will naturally dislodge with toenail growth. Engaging in activities involving heavy weights and wearing constrictive footwear and socks for an extended period within 6 months following surgery is contraindicated. Appropriate toenail trimming is imperative for daily care.

While the wedge resection and nail groove reconstruction with the hanging thread knot has demonstrated efficacy in treating onychocryptosis, further refinement is warranted. First, despite conducting a retrospective follow‐up study involving 155 patients, we did not establish a control group or perform statistical analysis. Second, to enhance the credibility of our treatment, it is imperative to enroll more patients and expand the evaluation criteria for therapeutic effects.

## 5 Conclusion

Effectively managing onychocryptosis necessitates a comprehensive approach, encompassing identification and resolution of the underlying etiology, excision of hyperplastic granulation tissue, partial resection of the nail bed, reconstruction of the nail sulcus to restore its normal anatomy, and correction of aberrant nail growth patterns.

## Author Contributions

Y.H. and X.Z. contributed to the conception and design of the study; Z.Z. and L.X. performed the experiments, collected and analyzed data; Y.H. wrote the manuscript; C.L. revised the manuscript. All authors reviewed and approved the final version of the manuscript.

## Ethics Statement

The current study was conducted in accordance with the Helsinki Declaration of the World Medical Association and approved by the Ethics Committee of First Affiliated Hospital of Chinese PLA General Hospital.

## Consent

Informed consent was obtained from all the study subjects before enrollment.

## Conflicts of Interest

The authors declare no conflicts of interest.

## Data Availability

The datasets generated and analyzed during the current study are available from the corresponding author on reasonable request.
